# Assessing anti-malarial drug effects *ex vivo* using the haemozoin detection assay

**DOI:** 10.1186/s12936-015-0657-8

**Published:** 2015-04-01

**Authors:** Maria Rebelo, Carolina Tempera, José F Fernandes, Martin P Grobusch, Thomas Hänscheid

**Affiliations:** Instituto de Medicina Molecular, Faculdade de Medicina de Lisboa, Av Prof Egas Moniz, Lisbon, P-1649-028 Portugal; Centre de Recherches Médicales de Lambaréné - CERMEL, Albert Schweitzer Hospital, Lambaréné, Gabon; Institut für Tropenmedizin, Universität Tübingen, Tübingen, Germany; Centre of Tropical Medicine and Travel Medicine, Amsterdam Medical Centre, University of Amsterdam, Amsterdam, The Netherlands; Instituto de Microbiologia, Faculdade de Medicina, Lisbon, Portugal

**Keywords:** Malaria, field trial, Anti-malarial sensitivity testing, Resistance, Flow cytometry, Haemozoin

## Abstract

**Background:**

*In vitro* sensitivity assays are crucial to detect and monitor drug resistance. *Plasmodium falciparum* has developed resistance to almost all anti-malarial drugs. Although different *in vitro* drug assays are available, some of their inherent characteristics limit their application, especially in the field. A recently developed approach based on the flow cytometric detection of haemozoin (Hz) allowed reagent-free monitoring of parasite maturation and detection of drug effects in culture-adapted parasites. In this study, the set-up, performance and usefulness of this novel assay were investigated under field conditions in Gabon.

**Methods:**

An existing flow cytometer (Cyflow Blue) was modified on site to detect light depolarization caused by Hz. Blood from malaria patients was incubated for 72 hrs with increasing concentrations of chloroquine, artesunate and artemisinin. The percentage of depolarizing red blood cells (RBC) was used as maturation indicator and measured at 24, 48 and 72 hrs of incubation to determine parasite growth and drug effects.

**Results:**

The flow cytometer was easily adapted on site to detect light depolarization caused by Hz. Analysis of *ex vivo* cultures of parasites, obtained from blood samples of malaria patients, showed four different growth profiles. In 39/46 samples, 50% inhibitory concentrations (IC50) were successfully determined. IC50 values for chloroquine were higher than 200 nM in 70% of the samples, indicating the presence of chloroquine-resistant parasites. For artesunate and artemisinin, IC50 values ranged from 0.9 to 60 nM and from 2.2 nM to 124 nM, respectively, indicating fully sensitive parasites.

**Conclusion:**

Flow cytometric detection of Hz allowed the detection of drug effects in blood samples from malaria patients, without using additional reagents or complex protocols. Adjustment of the initial parasitaemia was not required, which greatly simplifies the protocol, although it may lead to different IC50 values. Further investigation of set-up conditions of the Hz assay, as well as future studies in various settings should be performed to further determine the usefulness of this assay as a tool for rapid resistance testing in malaria-endemic countries.

## Background

In the last decade, the number of malaria deaths has decreased in large part due to the availability of effective treatments, in particular artemisinin combination therapy (ACT) [1]. However, these achievements are in danger and might even be reversed because parasites with prolonged parasite clearance times (PCT), observed in patients treated with ACT, have emerged in Southeast Asia [2,3]. Indeed, this is considered an early sign of the development of parasite resistance [2,3] and a major concern in the fight against malaria, as illustrated by the WHO Global Plan for Artemisinin Resistance Containment issued in 2011 [4]. Artemisinin resistance, currently defined as prolonged PCT, has spread across Southeast Asia [5]. Recently, a Vietnamese patient who apparently acquired malaria in Angola failed to respond to intravenous artesunate/clindamycin and an oral ACT after returning to Vietnam [6]. It is not unlikely that it will emerge in sub-Saharan Africa, and drug sensitivities should be monitored pro-actively. In this scenario, *in vitro* sensitivity assays may play a crucial role in the future. *In vitro* assays allow reducing host-related factors and thus, provide an objective insight into the intrinsic sensitivity of malaria parasites.

Several phenotypic and genotypic methods have been developed and tried for drug testing in the field [7]. Genetic resistance markers are known for some anti-malarial drugs, but are not yet able to predict sensitivity to all commonly used anti-malarial drugs [8]. Only recently, alterations in the *kelch13* gene were linked to delayed parasite clearance in artemisinin-treated patients [9]. Thus, phenotypic assays continue to be important for detection of resistance and validation of genetic markers. The main phenotypic assays successfully used to detect drug resistance in the field include: (i) the microscopic schizont maturation test [10]; (ii) the incorporation of radioactive hypoxanthine [11]; (iii) ELISA assays for detection of pLDH [12] and HRP2 [13] antigens; and, (iv) fluorescent-based techniques using either fluorometry [14] or flow cytometry [15] to detect parasite DNA/RNA. However, inherent limitations are common, especially during field applications. The supply, handling and disposal of radioactive isotopes are major obstacles. Microscopy is labour-intensive and subjective, although it has a rather quick turn-around time (24–30 hrs) when compared to other techniques, especially ELISA-based methods, which can take up to 72 or even 96 hrs [16,17]. Moreover, assays may require the use of, often, expensive antibodies or DNA/RNA stains, highlighting the issues of adequate storage and cold chain as well as limited shelf life.

Regarding flow cytometry, the majority of cytometric methods apply combinations of dyes to reliably detect infected red blood cells (iRBC), which implies a complex multiparameter analysis [15,18]. Ideally, if parasite maturation was detectable using a direct and simple measurement of a product from the parasite, the need for additional reagents would be avoided.

Haemozoin (Hz) is produced in increasing amounts by the parasite as it matures inside the iRBC, constituting an optimal maturation indicator [10]. Measuring Hz with a simple flow cytometry method allows detection of parasite maturation and drug effects as early as 18 hrs after incubation in culture-adapted laboratory strains [19].

The objectives of this study were to evaluate if the Hz detection assay could be easily set up in a remote malaria-endemic area, and to assess whether anti-malarial drug effects could be detected in wild-type strains obtained from malaria patients, using a simple protocol.

## Methods

The study was carried out at the Centre de Recherches Médicales de Lambaréné (CERMEL) in Gabon, a malaria-endemic region in Africa. Ethical approval was obtained from the Institutional Review Board of the Medical Research Unit (CERMEL) of the International Foundation of the Albert Schweitzer Hospital.

### Samples

EDTA anti-coagulated blood samples from malaria patients were obtained from the Clinical Analysis Laboratory of the Albert Schweitzer Hospital after the samples had been processed for full blood count (FBC). Malaria diagnosis and parasite loads (number of parasites/μl of blood) were determined by standard microscopic observation of Giemsa-stained thick blood films. Briefly, parasitaemia was quantified by counting the number of parasites per microscopic field from a defined volume of blood (10 μl) spread on a defined area (1.8 cm2), as described elsewhere [20]. RBCs from these blood samples were washed twice in culture medium before further use.

### Flow cytometric detection of depolarized side-scattered light

**Figure 1:**
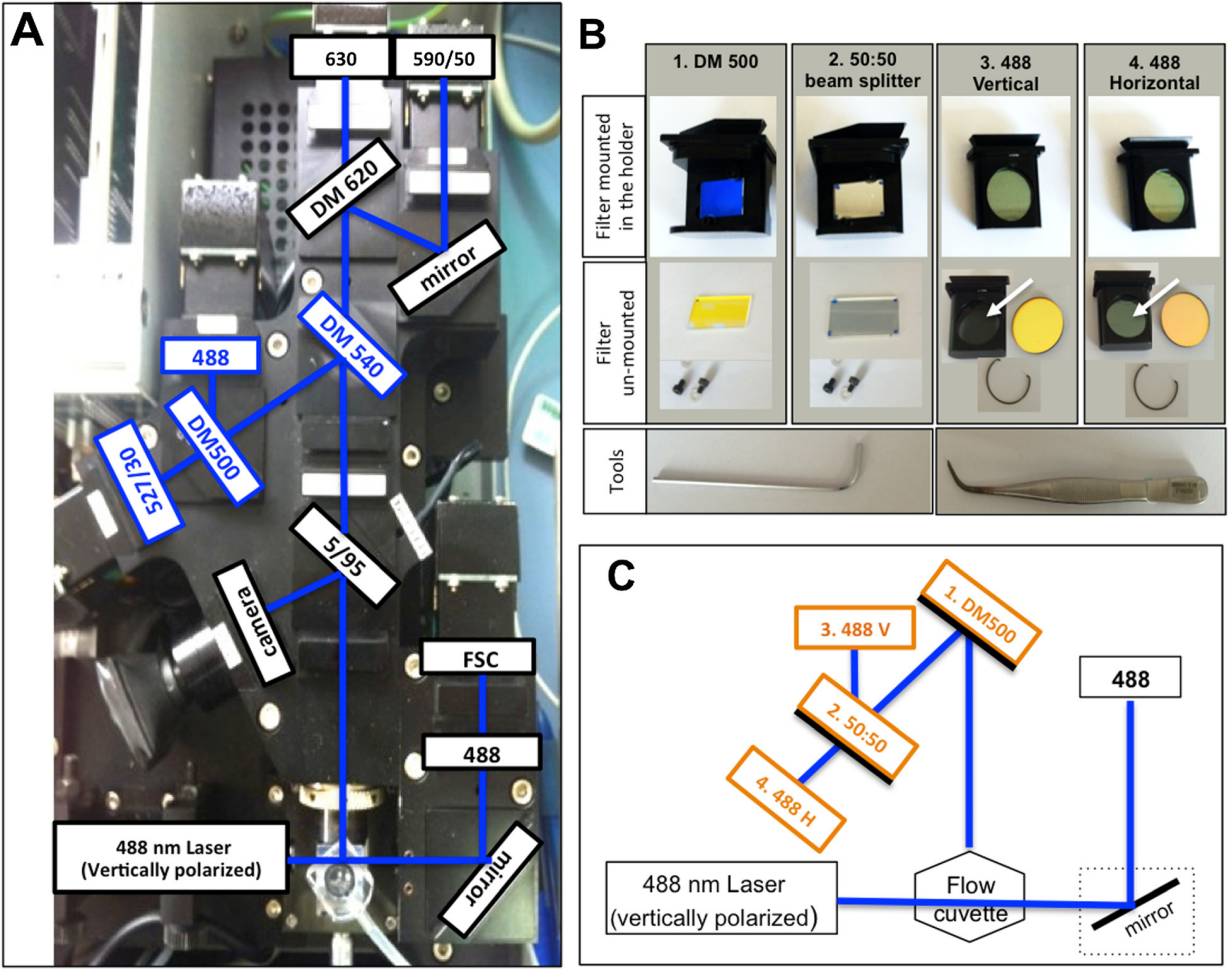
**Flow cytometry modifications to detect light depolarization.** The optical set-up of the Cyflow (Partec, Münster, Germany) **(A)** was easily modified to allow the detection of light depolarization. Four optical components from the original set-up (blue boxes in **A**) had to be replaced by the optical components shown in **B**, as described in the Methods section. Filters were simply replaced by unscrewing the original ones from the filter holder, or by removing the metallic ring (second row in **B**). This was accomplished using simple tools (bottom row in **B**). Note that in this case, polarization filters were glued inside the holder (arrows in **B**) so that they would not move, since to attain the best depolarization signal polarizers should be perpendicular to each other. The final optical layout to detect light depolarization is presented in C.

Flow cytometric analysis was performed using a CyFlow® Blue (Partec, Münster, Germany) available on site. The existing configuration, which was modified on site for this study and is shown in Figure 1, consisted of forward scatter (FSC), side scatter (SSC) and three fluorescent detectors (FL1, FL2 and FL3). A set of four optical filters was necessary, as shown in Figure 1B: (1) a 500-nm dichroic mirror; (2) a 50:50 beam-splitter; (3) a 488-nm vertical polarizer; and, (4) a 488-nm horizontal polarizer. Briefly, a 500-nm dichroic mirror (DM) (B1) was placed on the site of the original 540-nm DM and the other original 500-nm DM was replaced by a 50:50 beam-splitter (B2), which allowed the creation of two SSC detectors (Figure 1). A 488-nm filter coupled with a polarizer in the same orientation as the incident laser beam (vertical) was placed in front of one of the SSC detectors. Another 488-nm filter coupled with a polarizer perpendicularly orientated in relation to the laser beam (horizontal) was placed in front of the other SSC detector, allowing the detection of light depolarization (Figure 1C). The optical components required to modify the optical bench of flow cytometers can be obtained directly from the instruments’ manufacturer.

### Anti-malarial drugs

Samples were tested against different concentrations of chloroquine, artesunate and artemisinin (Sigma Aldrich, St Louis, MO, USA). Stock solutions of chloroquine were prepared in sterile water, and artemisinin and artesunate were prepared in pure methanol. Doubling concentrations, ranging from 25 to 200 nM for chloroquine and from 0.12 to 128 nM for artemisinin and artesunate, were prepared from the stock solutions in in complete malaria culture medium (CMCM), which consists of RPMI 1640 supplemented with 25 mM HEPES, 2.4 mM L-glutamine, 50 μg/mL gentamicin, 0.5% w/v Albumax, 11 mM glucose, 1.47 mM hypoxanthine and 37.3 mM NaHCO3.

Each drug concentration was tested in triplicate.

### Haemozoin detection assay

RBCs obtained from malaria patients were diluted at a haematocrit of 5% in CMCM. To simplify the assay, the parasitaemia was not adjusted so that, eventually, the use of uninfected blood could be avoided. A volume of 100 μL was distributed into the wells of a 96-well plate, previously loaded with 100 μL of anti-malarial drugs at different concentrations, or 100 μL of CMCM for the drug-free controls, respectively. Plates were incubated for 72 hrs at 37°C in 5% CO_2_ atmosphere. Flow cytometric measurements were performed at 24, 48 and 72 hrs of incubation. Parasite maturation from ring-stage to schizonts was assessed based on the increase in the percentage of Hz-containing cells overtime, as described before [19]. To assess parasite replication (re-invasion), the same samples were stained with SYBR green I at 1x (Invitrogen, Carlsbad, CA, USA), as described elsewhere [19].

### Histidine-rich protein-2 (HRP2) enzyme-linked immunosorbent assay (ELISA)

For the HRP2-ELISA, RBCs were diluted in CMCM at a haematocrit of 3%. Parasitaemia was adjusted to 0.05% using RBCs obtained from healthy volunteer donors. A volume of 100 μL was distributed into the wells of a 96-well plate, previously loaded with 100 μL of anti-malarial drugs at different concentrations or 100 μL of CMCM for the drug-free controls. Plates were incubated for 72 hrs at 37°C in 5% CO_2_, after which they were frozen at −20°C until the HRP2-ELISA was performed according to standard procedures [21].

### Data analysis

**Figure 2:**
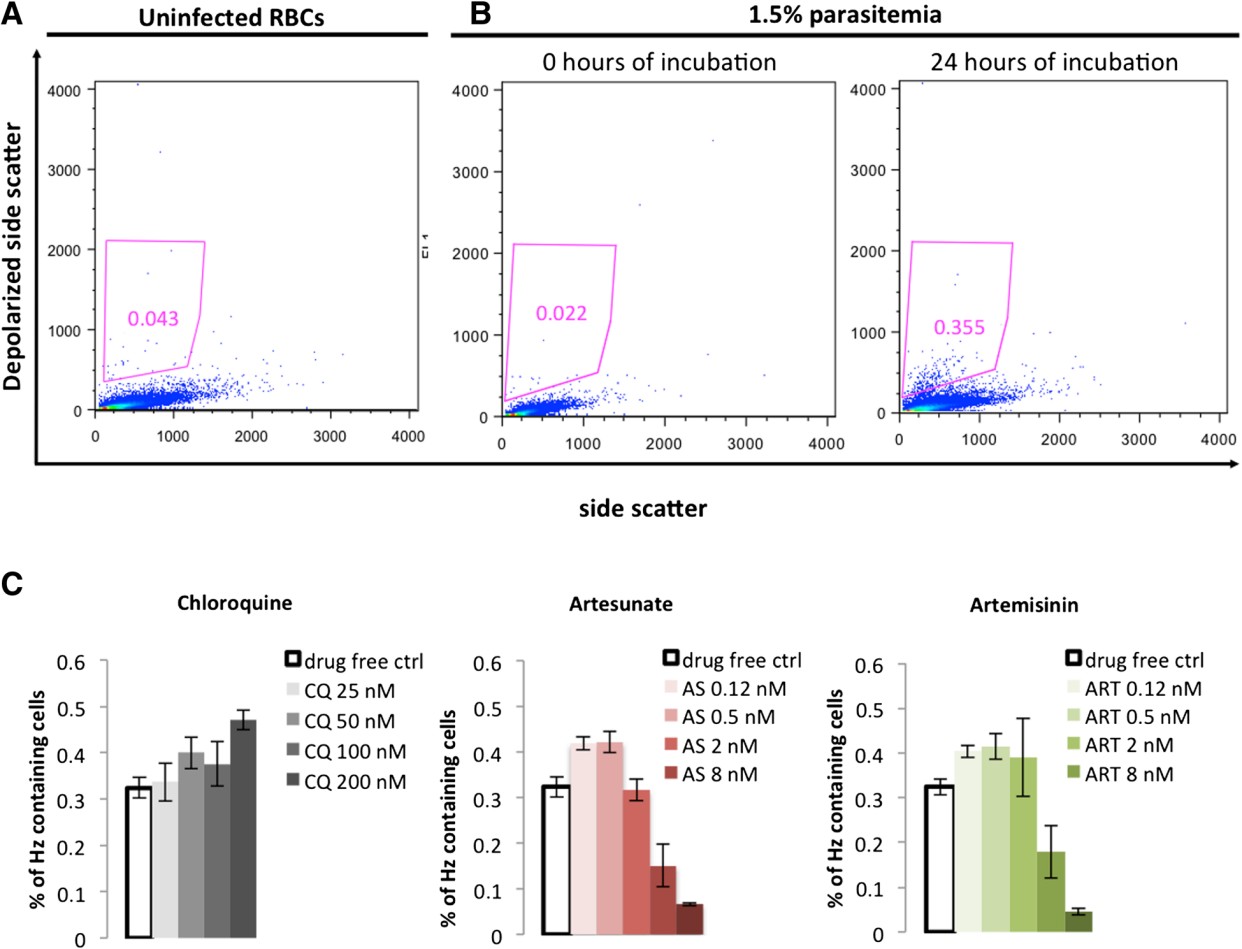
**Representative analysis of drug effects assessed after** ***ex vivo*** **culture of blood samples from malaria patients.** Representative plots of flow cytometric analysis of an uninfected blood sample **(A)** and a sample from a malaria patient with 1.5% parasitaemia **(B)**. At time point 0 of incubation, no difference in depolarizing events is observed between the infected samples and the uninfected control **(A and B)**. However, Hz is produced as the parasite matures, and after 24 hrs of incubation an increase in the percentage of depolarizing cells is detected **(B)** from 0.02 to 0.36. In this example, drug effects could be determined after only 24 hrs of incubation **(C)**. Contrary to chloroquine, where resistance was observed, artesunate and artemisinin are still effective drugs **(C)**.

Flow cytometry results were analysed using FlowJo software (version 9.0.2, Tree Star Inc., Oregon, USA). Depolarizing events were defined in plots of SSC *versus* depolarized-SSC, as those with a signal above the background observed in the uninfected control (gate in Figure 2A and B). To determine SYBR green I-positive cells, green fluorescence (FL1) *versus* red fluorescence (FL3) plots were used. The FL1 detector had a 527/30 band-pass filter and FL2 had a 620-nm long-pass filter. SYBR green I-positive events were established based on a stained uninfected control and had to be adjusted at each time point, always using the uninfected SYBR green I-stained sample from the corresponding time point. A non-linear regression model (sigmoidal dose–response/variable slope) was used to calculate the individual 50% inhibitory concentrations, with SigmaPlot-Systat Software (Chicago, IL, USA). Only those samples with a ≥2 ratio of drug-free control to highest drug concentration were included.

## Results and discussion

### Growth and maturation of wild-type *Plasmodium falciparum* strains

Forty-six samples from malaria patients were analysed during this study. Parasite loads ranged from 50 to 452,000 parasites/μL of blood (median of 15,000 parasites/μL). *Ex vivo* cultures of infected RBCs showed that parasites had four different growth profiles (Table): (i) seven samples showed no maturation, as defined by an increase in depolarization; (ii) another eight samples showed maturation at 24 hrs and replication at 48 hrs; (iii) 17 samples showed maturation at 24 hrs but no re-invasion occurred; and, (iv) 14 samples had a delay in parasite growth, with maturation being observed at 48 hrs and replication at 72 hrs.

One crucial step in the *in vitro* sensitivity assays is the culture of parasites [22]. Maturation of *Plasmodium falciparum* from early rings to late schizonts takes 42–48 hrs *in vitro* [23] and consequently, an increase in parasitaemia can only be observed every 42–48 hrs, after re-invasion of RBCs occurs. Differences in *ex vivo* parasite maturation and replication have already been observed in strains obtained directly from different patients [24,25]. Indeed many factors related to the protocol, the host and the parasite itself might greatly influence the *in vitro* growth of parasites.

Regarding the protocol factors, such as the type of anticoagulant used to collect the blood from the patients to the atmosphere where the parasites will be incubated, have to be taken into account [7]. During this study EDTA-collected blood was used. Although the use of EDTA is discouraged by the reference protocol from MR4 [26] it has been shown by different studies that EDTA-collected blood can be successfully used for *ex vivo* drug testing [27-31]. In one of these reports even long-term cultures of parasites present in patients’ blood were accomplished [31]. Moreover, the use of specific anticoagulants requires drawing more blood just for the purpose of sensitivity testing. This can be avoided by using EDTA-anticoagulated blood, which was obtained as part of the routine FBC analysis, preventing all inherent problems associated with an extra blood drawing.

The incubation atmosphere recommended for *P. falciparum* growth in culture include the use of a low O_2_ atmosphere [26]. However, such mixed gas atmospheres may not be available in the resource-limited settings found in malaria endemic countries. Because of this, it has been investigated whether a simple 5% CO_2_ atmosphere could be used instead without compromising parasite survival after drug treatment [32]. Results showed no differences in parasite survival between trigas (5% CO_2_, 5% O_2_, 90% N_2_), candle jar or a 5% CO_2_ atmosphere [32].

Undoubtedly, host-specific factors, often difficult to control, ranging from the immune response to the presence of pharmacologically active substances might influence the growth of the parasite *in vitro.* Indeed, the fact that some of the patients might already have been treated at the time of blood collection during this study cannot be discarded, possibly explaining some of the differences observer in the parasites’ growth profiles. Yet, it is very difficult to control for all these factors and this might not only imply detailed histories, but eventually laboratory test to confirm immune status or presence of drug-metabolites. Perhaps explaining why very few studies on this filed are including such detailed information.

Conversely, parasite-related factors may even be more important. The delay between sample collection and processing influences the viability of freshly collected clinical isolates, because it is considerably decreased after a sample has been kept for several hours at room temperature [7]. However, in one study where all 43 samples were cultured within 30 minutes of collection [25], 50% developed into schizonts within 27 hrs, while the other half reached schizont stage only between 28 and 63 hrs. In this study no correlation between the delay until culture and any of the four parasite growth patterns was observed, supporting the idea that possible host factors may have been more relevant.

### Detection of anti-malarial drug effects by the haemozoin assay

In *P. falciparum-*infected patients the majority of circulating iRBC are ring forms that contain little or no detectable Hz, as observed in a previous report whereby flow cytometric assessment of Hz these forms could not be detected [33]. This study confirms this observation, as at 0 hrs, no difference in depolarizing events was observed between the infected and the uninfected samples (Figure 2A and B). Only after incubation the percentage of depolarizing cells increased, indicating higher Hz content and, thus maturation (Figure 2B), seen in drug-free controls or drug-resistant parasites. This contrasts with diminished or absent depolarizing events when anti-malarial drugs are effective (Figure 2C). Drug inhibitory effects were determined in 39/46 (85%) of samples, in which parasite maturation was observed (groups 2, 3 and 4). In 25 samples (groups 2 and 3), drug effects were measurable at 24 hrs, as expected from culture-adapted strains [19]. In the remaining 14 samples (group 4) parasite maturation was delayed; however, it was still possible to detect drug effects at 48 hrs of incubation.

### IC50 values of chloroquine and artemisinins

**Figure 3:**
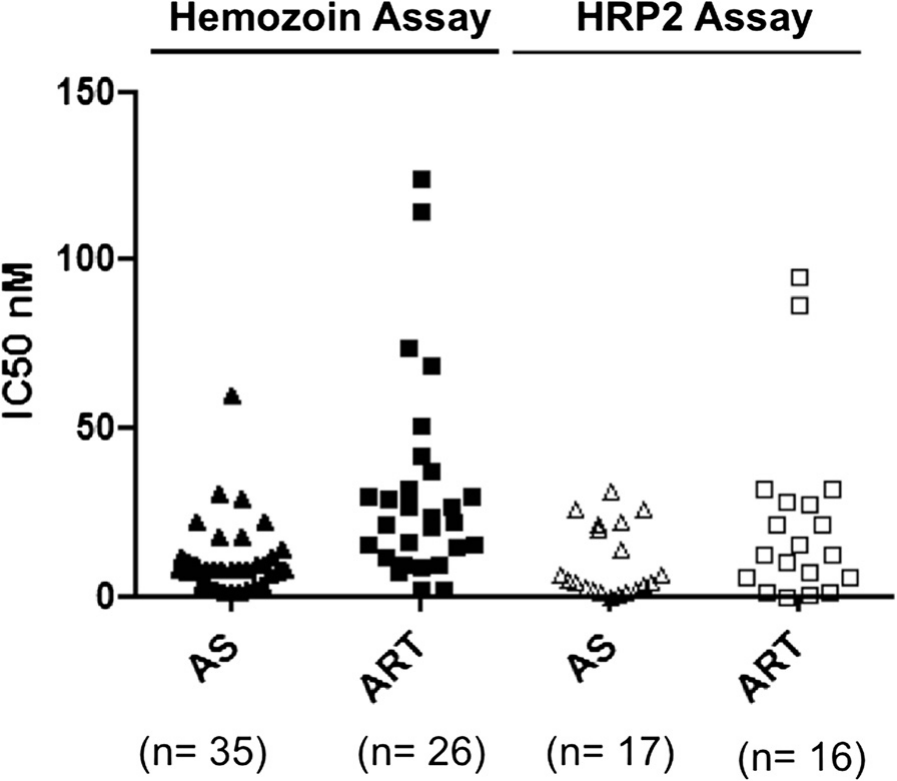
**Inhibitory 50% concentrations obtained for artesunate and artemisinin.** Using the Hz detection assay, IC50 values for artesunate and artemisinin differed between samples ranging from 0.9 to 60 nM and from 2.2 nM to 124 nM, respectively. IC50 values obtained by the HRP-2 ELISA ranged from 0.6 to 31 nM for artesunate and from 0.6 to 94.8 nM for artemisinin.

The IC50 values are shown in the Table 1. There was a poor correlation between the IC50 values of individual samples and the different assays. In fact, this is commonly observed even when using the same laboratory-adapted strain [19]. Indeed, several factors, such as the initial parasitaemia, the haematocrit and the end point for measuring parasite growth can influence IC50 values [7,34]. Samples with higher initial parasitaemia (group 3), showed increased IC50 values for artesunate and artemisinin (Table). Apparently, a higher initial parasitaemia may be associated with an increase in inhibitory drug concentration of artemisinin, artesunate, chloroquine, and mefloquine [11], which could explain some of the increase observed in the IC50 values. Interestingly, mean IC50 values for artesunate and artemisinin in isolates that had a delayed growth (group 4) were somewhat lower than in groups 2 and 3, where maturation was detected at 24 hrs. This could be due to the different growth profile of drug exposed and non-exposed parasites in group 4. Overall, IC50 values ranged from 0.9 to 60 nM and from 2.2 nM to 124 nM for artesunate and artemisinin, respectively (Figure 3). These values are higher than previously described in the same region, using the HRP-2 assay [35]. Because several assay-related factors can influence IC50 values they may not be directly comparable between different assays [7,34]. In the previous study, the final haematocrit was 1.5% [35], while in the Hz detection assay it was 2.5%. It has been observed that higher haematocrits may cause an increase in IC50 values [34]. Preliminary data using the same culture-adapted strain showed that the IC50 value for dihydroartemisinin increases from 1.7 nM to 7.5 nM when the haematocrit of the sample is raised from 1 to 2.5% (unpublished data).

**Table 1:** **Summarized data of isolates exhibiting different** ***ex vivo*** **growth profiles analysed by the haemozoin assay**

	**Group 1**	**Group 2**	**Group 3**	**Group 4**
Growth profile	No maturation	Maturation at 24 hrs and replication at 48 hrs	Maturation at 24 hrs but no replication	Delayed maturation
Number of samples	7	8	17	14
Parasitaemia	0.01%;	0.3%;	3.5%;	0.3%;
(median; range)	0.001-0.1%	0.2-2.2%	0.2-12%	0.1-1.9%
IC50 Artesunate (mean)	nd	10.5 nM	15.2 nM	5.6 nM
IC50 Artemisinin (mean)	nd	46 nM	47.3 nM	14.4 nM

nd – not determined.

IC50 – 50% inhibitory concentration.

Note: Results from chloroquine were not presented in this Table because the majority of the samples (32 out of 46) had IC50 values higher than 200 nM. Chloroquine had an inhibitory effect in only four samples from group 3 (IC50 mean = 76.8 nM). In ten samples, chloroquine activity could not be determined.

For chloroquine, 70% of the samples had IC50 values higher than 200 nM, indicating resistance, which is in line with an earlier reports and which is explained through the high usage of amodiaquine, many years after the use of chloroquine had been curbed and finally abandoned [36,37]. In Malawi [38,39], Kenya [40] and Tanzania [41], chloroquine resistance decreased after its withdrawal, contrary to the study site, even though chloroquine treatment was discontinued 11 years ago, as observed by others [37,42]. One explanation brought forward is the use of artesunate/amodiaquine, because amodiaquine appears to select mutant *pfcrt* allele, which is responsible for chloroquine resistance [43].

Interestingly, the Hz-detection assay showed in some of the chloroquine-resistant strains that the percentage of Hz-containing cells increased as chloroquine concentration increased as well (Figure 2C). It is known that several drugs, specially quinoline-type drugs, directly interact with Hz, as discussed elsewhere [44]. This interaction may lead to alterations in the crystals distribution within the parasite that may affect the depolarized light signal. Recently it has been shown that parasites containing several small but distributed Hz crystals can have a higher depolarized signal than a parasite containing a single large clump of Hz [45]. In this context it is not unlikely that at higher concentrations of chloroquine some of the drug may interact with Hz avoiding its coalescence. Thus, explaining why at higher concentrations of this drug the signal seems to be increased. Certainly, the mechanism of this observation is not known, and further investigation is required to understand the exact cause of this phenomenon.

During this study the HRP2-ELISA was performed alongside. However, drug effects could only be detected in 17 samples (37%), with IC50 values ranging from 0.6 to 31 nM for artesunate and 0.6 to 94.8 nM for artemisinin (Figure 3). This success rate appears to be at the lower end of reported studies with 45% [14], while others had success rates of 75% [46] and 87% [13]. Of note, these samples were collected on purpose to be used exclusively in the scope of these studies. Contrary to this, here, samples were collected for other purposes, which might have contributed to a lower success rate. Furthermore, samples were preselected based on their parasitaemias of 0.01% or higher [13], while in this study all samples were included. Apparently, re-invasion of uninfected RBCs (replication) is considered the main criterion for the success of the HRP2 assay [21]. However, even when no schizont maturation is observed after 24 hrs of incubation, samples can still be successfully tested by the HRP2 assay [21]. During this study, most results (14 out of 17) were obtained in samples that exhibited delayed-growing parasites (group 4), whereas drug effects were only detected in two samples from groups 1 and 2, and in five samples from group 3.

Indeed, the low detection limit is a major advantage of the HRP2 assay. For instance, the Hz assay failed to determine drug effects in samples from group 1, possibly due to the low parasitaemias present in this group, which were below the previously reported 0.3% detection limit of the Hz assay [19]. On the other hand, when parasitaemias are higher than 0.1% [21], samples have to be diluted with uninfected RBCs from healthy donors, which can be a limiting step. Results from group 3, where the parasitaemia ranged from 0.2 to 12%, indicate that the Hz detection assay does not seem to require adjustment of the parasitaemia to allow the detection of drug effects.

### Field applications of flow cytometry

The optical bench of the CyFlow® flow cytometer available on site had a typical configuration common in most small instruments, consisting of one blue-laser (488 nm) and detectors for FSC, SSC and three fluorescences FL1 (green), FL2 (orange) and FL3 (red) (Figure 1A). For the detection of light depolarization the original set-up was easily modified by simply changing the respective filters and mirrors, even taking advantage of the existing filter holders (Figure 1B). The optical bench layout required for the detection of light depolarization is simple (Figure 1C) and therefore, other instruments should also be easily modifiable, unless they use fiber-optic cables for light collection.

Few studies describe the use of flow cytometry for drug testing in the field [15,47], possibly because of the perceived expense and complexity in setting up and running such instruments. Nowadays the number of simple, robust and portable flow cytometers has increased, including instruments such as the Attune® (Life Technologies, Carlsbad, USA), the Accuri™C6 (BD Biosciences, La Jolla, USA), the Cyflow Cube 6 (Partec, Münster, Germany), among others. It has been shown that theses instruments can be easily modified to detect Hz-caused light depolarization [45] and most of them can be used with an autosampler for higher throughput [48]. Moreover, the initial purchase costs of these instruments have dropped substantially from those practiced before for larger instruments, allowing them to be used and available in the field. Indeed, recent field studies take advantage of this, by using for example the Accuri C6 [15,47]. Even smaller and simpler instruments used for CD4+ T cell counting in HIV-infected patients exist, such as the CyFlow®miniPOC from Partec, which are used in low-resource settings [49]. Although the way was often difficult, cytometry is no longer the very expensive, high-end technology based on bulky instruments. In the future, simple image cytometers might replace flow cytometers, as they seem to perform a broad range of measurements, and eventually parameters such as light depolarization could be detected as well [50,51].

### Opening new avenues for anti-malarial drug testing in the field

This study showed that drug effects of clinically relevant anti-malarial drugs as well as resistance to chloroquine could be assessed by simply detecting Hz. This method measures parasite maturation and does not require re-invasion to occur; consequently, results can be obtained earlier than with other currently available methods, except for microscopy. However, microscopy relies on trained observers’ ability to detect morphological changes of iRBC [52], which sometimes can be rare. Flow cytometric measurements can provide more objective, reliable and effective results than microscopy, as it has been previously observed in a different context [53]. Moreover, it allows the assessment of additional parameters, such as DNA and RNA content, which can improve parasite detection [51]. Finally, drug effects could be detected without having to decrease the sample’s parasitaemia. This may greatly simplify the protocol as it avoids the need to obtain blood from healthy donors. Yet, this seems to lead to increased IC50 values. Thus, whether parasitaemia ought to be adjusted or not should be further investigated.

These findings open new avenues for other Hz-detection methods. Interestingly, several Hz detection methods exist [54-58] and they could possibly be used to detect drug effects as early, or even earlier, than the flow cytometric Hz detection.

## Conclusion

This study conducted in the field showed flow cytometry could be easily implemented and performed in field conditions. Flow cytometric detection of Hz could be used as an alternative tool to assess drug effects on parasites obtained directly from patients’ blood samples, without the need for additional reagents or complex protocols. However, further optimization of the Hz assay regarding its set-up conditions, for example, changing the haematocrit, may contribute to obtain IC50 values more comparable to the ones that have been previously reported [13,59].

Future studies should be performed in various settings, to further investigate the Hz assay and its usefulness as a tool for rapid resistance testing in malaria-endemic countries.
